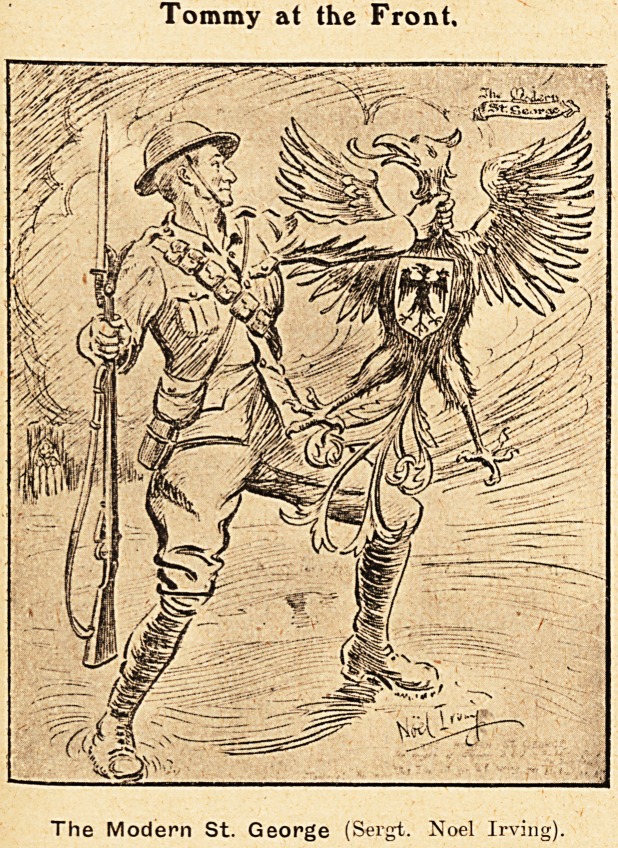# Hospital and Institutional News

**Published:** 1917-12-15

**Authors:** 


### HOSPITAL AND INSTITUTIONAL NEWS.

### Tommy at the Front.

The Modern St. George (Sergt. Noel Irving).

### HOSPITALS AFTER THE WAR.

As we anticipated, the Minister of Pensions has appointed a committee, consisting of Sir Arthur Newsholme, Chief Medical Officer of the Local Government Board, Dr. Smith Whittaker, and Dr. Meredith Richards, deputy-chairman of the English and Welsh Insurance Commissions, together with a representative of the War Office, to ascertain the accommodation in civil hospitals in Great Britain. The Committee held its first meeting on the 6th instant, the intention being to report at an early date on the various aspects of the investigation. Presumably this Committee will recommend what they consider to be the best course to adopt to provide institutional treatment and accommodation for men disabled through the war, whom it has been decided not to admit to military hospitals, nor, and properly, to send to Poor-Law institutions. At the present time the accommodation at many general hospitals is fully taxed, the waiting-lists are heavy, and it is a most urgent matter to decide how and where to provide the accommodation for the disabled warriors in question. From a national point of view it is absolutely essential that the men who have been disabled through the war should promptly and continuously receive the institutional treatment which each may require. Having regard to the momentous and grave issues involved, the Minister of Pensions will be well advised to strengthen his Committee.

### DOES A HOSPITAL SOCIAL SERVICE DEPARTMENT PAY?

In the annual report of the Samaritan Fund, St. Thomas's Hospital, the above question, which is constantly raised, has been answered for the first time. The paragraph is worthy of repetition: "As in preceding years," it runs, "a great many inquiries have been received from other institutions about the Social Service Department at St. Thomas's Hospital, and in particular one question is constantly asked—namely, 'Does it pay?' Estimating, roughly, that the cost of treatment is 5s. per head, and that 4,744 were sent back either to private doctors, panel doctors, or Poor-Law authorities, this year it may be assumed that nearly £1,200 has been saved. In addition to this, £2,179 was handed over to the funds of the hospital, representing patients' donations and maintenance charges collected from Insurance Committees and County Councils, part of which is directly due to the work of almoners. But it cannot be too strongly emphasised that the treasurer and governors of the hospital did not institute the system with any view to financial gain, but wholly to ensure that the benefits offered by hospital treatment should be directed to the right channel, and, furthermore, that no effort should be spared to ensure that those whom the hospital accepts as patients shall have every possible opportunity to benefit by that treatment. The governors considered that wastage of skill, care, and effort was bound to exist without a Social Service Department to extend the hospital treatment and influence into the homes and lives of the patients. On these grounds alone can the work of the Social Service Department of a hospital be justified. . . ." Considering the large outlay on all the most modern forms of treatment, it can surely be claimed that "it does pay" to establish a Social Service Department, if through such means the work can be solidified and completed.

### A CHAIRMAN ON THE OUTLOOK.

Mr. G. H. Strutt, chairman of the weekly board of the Derby Royal Infirmary, has dealt lately with some of the difficulties ahead. In spite of an increase in the income from the Saturday Fund, the year has changed a balance in hand into a deficit of £700, and a general increase in the number of patients has accompanied "a seriously depleted and constantly changing staff." Internal repairs and renovations have been necessarily postponed, and Mr. Strutt foresees a rush of civilian patients on the declaration of peace. He believes that the high prices and high taxation will continue, and that the hospital will have to rely upon its ordinary resources to meet the heavy charges which will then have to be incurred. We see no way out of the difficulties, which are only typical, but in the organisation of sectional subscriptions from every class which the hospital serves.

### BABY WELFARE.

Lord Rhondda's great enthusiasm for child-welfare work during his tenure of the office of President of the Local Government Board is a matter of common knowledge, and now that his translation to the Food Controllership prevents him from proceeding with what he regarded as the most important piece of work to which he had ever put his hand, Lady Rhondda is doing her best to keep before the public the ideals to which her husband is so attached. Presiding on November 14 at a meeting of the Council of the Baby Week Exhibition, at which a scheme of reorganisation was adopted, Lady Rhondda emphasised the necessity for keeping the movement alive and not relaxing their efforts until the Bills necessary for the welfare of the babies and their mothers were passed by Parliament. Dr. Pritchard announced that the Baby Week Exhibition movement now comprised 90 societies and 600 local committees.

### SWANSEA ORPHAN HOME

At a special meeting of the governors of the Swansea Orphan Home on November 23, Mr. Roger Beck, one of the builders of the great South Wales industrial firm of Baldwins, made an offer to purchase the commodious mansion known as Brooklands as a permanent Home for the orphanage. It is now occupied by the Vicar of Swansea, and also used during the assizes as the Judges' lodgings. The governors readily accepted this generous and magnificent proposal, and undertook to maintain the new Home, which will undergo certain structural alterations to fit it for its purpose. The mansion is situated in its own grounds, and commands a fine view of Swansea Bay and Bristol Channel; it is freehold, and the capital value of the gift is estimated at about £10,000. It will be remembered that recently Mr. Beck negotiated with the Swansea Corporation for the acquisition of a plot of land upon which he proposed to build a new orphanage, but questions having been raised in the Council Chamber as to the future constitution of the governing body of the orphanage—the suggestion being that it was a Church of England affair—Mr. Beck, who is himself a member of the Society of Friends, promptly withdrew his offer rather than allow any sectarian differences to be introduced. Steps will now be taken, it is understood, to provide an adequate endowment for the new Home, and already Mr. Cory-Yeo has promised to subscribe £2,000 to that end.

### THE FRUITS OF CRITICISM.

The Ministry of Pensions has come in for its share of criticism, and on the whole this criticism should be welcomed, since Government Departments tend to lag behind public opinion. It is interesting to learn, therefore, that the Ministry has started a Publicity Bureau "to correct," in the words of Mr. Hodge, "the lies told about the Department." He quoted the condemnation of a judge, who described the Ministry as robbing a man of his pension because it gave a soldier who had been injured in an accident the choice between the damages offered to him by the tramway company and a continuance of the pension granted to him on its account. Mr. Hodge declared that it was unreasonable that he should be paid twice for the same injury. A committee was at work to survey the extent of institutional treatment available for all cases which needed it. So far, so good. If the delays and mistakes in the granting of adequate pensions are fewer than they were, the soldiers have to thank the criticism to which Mr. Hodge's Department has been subjected. It is better that the Department should suffer unfair comment than that any soldiers should suffer unfair delay.

### "TRUTH'S" CHRISTMAS NUMBER.

The worst of being a journalist with a reputation for originality is that each success is harder to repeat than the last. A friendly contemporary, therefore, opens each successive Christmas number of *Truth* with an anxious expectancy. The latest Christmas number has a genuine novelty by the inclusion of a facsimile of a daily newspaper which is a riot of caricature, from the huge headlines to the smallest detail. The type chosen for the presentment of this enormous joke makes its object unmistakable, and it is certainly excellently done. There was once on sale in London a paper entitled *The Daily Liar*, and its non-success was presumably due to a natural confusion between it and other journals. *Truth's* joke is much better than the paper or papers which it satirises, and we believe that "The Daily Stunt," as the satire is called, will be more popular than anything else in a bulky Christmas number. There are, besides *Truth's* own proper pages, two coloured pictures presenting the Kaiser's nightmare and a grim row of gibbets, from which the hostile Emperors and the German Crown Prince hang in a row. This is entitled "A League of Peace"—a thing fiercer than any drawing by Raemaekers.

### BRITISH TROOPS IN DURBAN.

Durban has become very popular with the British troops passing through. Not only is it a beautiful and interesting town, but it is a remarkably hospitable town, and soldiers are so kindly treated that the Natal seaport has earned almost a world-wide reputation for hospitality. For this, especial credit is due to the ladies of Durban and to the municipal authorities. The soldiers are given the free use of the trams, and the service is excellent and extensive. They are also given the free use of the large Ocean Beach open-air swimming bath and bathing enclosure, both of which are close to the military camp. A splendid work is being carried on in the centre of the town at the Wesley Hall in West Street, under the supervision of Captain the Rev. G. H. P. Jacques, military chaplain, and there a quarter of a million soldiers have, up to the present, been entertained and given free refreshments. There are also three or four large "huts," generally crowded with men, where provision is made for the convenience and recreation of the soldiers, and all food and drink—a cup of tea, a piece of cake, or an egg—cost the same price, one penny. There are also two free Army and Navy Institutes where billiards are provided in addition to reading and writing rooms. The ladies of Durban take a pride in their patriotic personal service, and in providing foodstuffs and collecting money. Saturday street collections in aid of various war relief funds are a regular weekly institution, and one that always meets with generous response. No wonder British soldiers leave Durban with pleasant memories.

### THE COST OF A NURSE.

THE Central Council for District Nursing in London has informed the London County Council that the remuneration given by the Council for the services of a whole-time nurse does not meet the necessary expenses, which would appear in the majority of instances to be from £120 to £130 per nurse, and the Central Council asks that the matter may receive the sympathetic consideration of the County Council. The agreement between the County Council and the local nursing association provides that the association shall supply a nurse, who attends at the treatment centres daily at specified times, and that the association shall arrange for visits to be paid by the association's nurses to the children's homes where necessary. In return for these services the Council makes a payment of £100 a year, which is to cover the salary of the nurse, her travelling expenses, and the provision of drugs and bandages required for the treatment at the children's homes. In view of the general increase in the cost of living, the County Council admits that some increase in the rate of remuneration is justifiable, and it has raised the amount to £115 per annum. There are forty full-time district nurses engaged, or proposed to be engaged, in the treatment of minor ailments.

### THE COMBATING OF VENEREAL DISEASES.

THE social history of the present day, when it comes to be written, will recognise that two new influences have been brought to bear on the problem of venereal disease. The first of these is the practice of open speech in the place of a purposeful silence; the second the suppression of the advertising quack. Upon the one it has been possible to found a public educational campaign; and upon the other, subsidised facilities for early and effective treatment. Both of these policies are really striking departures from traditional customs, and the National Council formed for the purpose of combating venereal diseases has a right to feel that it has by no means worked in vain. The second annual report of the Council shows that there is still educational work to accomplish. Not all local authorities have established public opportunities for treatment, and some of the defaulters are found at certain of the seaport towns, where notoriously there is special need for such opportunities. As a consequence the provisions of the Act relating to the abolition of the unqualified treatment of venereal diseases cannot be put into operation. Again, it seems that even yet there are hospitals which refuse treatment to patients suffering from the early stages of these diseases, presumably under the influence of the view that to treat the offender is to encourage immorality. Public opinion is the only force which can be relied on in these two instances, and the Council exists to create this opinion. Another educational opportunity is afforded by the rising generation, which is ever coming into the danger zone and deserves the knowledge necessary for self-protection. Organised committees in various parts of the country, lectures, and pamphlets are the machinery mostly employed, and by these warning and help must certainly be afforded. Education and early treatment are thus secured their chance, and it may be hoped that good results will follow. The practical test will be afforded by time, which alone can speak the authoritative word. What has been gained so far is the establishment of methods which may reasonably be expected to be useful, and the verdict of experience will say how far the anticipation is well-founded.

### SHOULD A HOSPITAL TELL?

IN the matter of treating ex-soldiers at hospitals an interesting point arises as to whether a hospital should respond to a request from a Local Pensions Committee to state exactly what the pensioner is suffering from. In some cases, the man's disability may have been aggravated by, if it has not originated entirely in, venereal disease. Yet, with a not unnatural generosity, the medical officer who has filled up the man's discharge papers may have slurred over that aspect of the matter, and the pensioner may be officially suffering from "cystitis," "prostatitis," or "stricture." It is a difficult matter to decide whether the hospital should furnish the Local Committee with an exact certificate (which may adversely affect the man's pension), or whether it should allow the ambiguity of nomenclature to continue. A stern sense of public duty would seem to call for an exact statement of the facts, but human sympathy with a man who has been fighting for his country generally secures for him that procedure which will be most beneficial to him.

### COMPULSORY RATIONING IN GERMANY.

A WRITER in the latest number of the *National Food Journal* throws much light on the working of the food-ticket system in Germany. He deals with both legitimate and illegitimate methods of obtaining tickets, or rationed food without tickets. It appears that one method is to frequent the back-doors of public-houses favoured by the fraudulent ticket dealer and his agents. To obtain food itself without the incumbrance of a ticket a plan is to take an excursion train to a farming district, leaving town with a full purse and an empty portmanteau, and returning with empty pockets and well-stocked luggage. But these methods are both expensive and risky; not long ago a two days' auction was held at a German railway station of food discovered in passengers' luggage, which its owners had found it advisable to jettison. The article contains a word of caution which may or may not be intended to prepare us for more tickets. The writer states that the German ticket system has undoubtedly saved the nation from early defeat in the war by reducing consumption to a minimum far below any that voluntary effort could have secured.

### WIGAN'S NEW SUPERINTENDENT.

Mr. Hedley Lucas, whose appointment as secretary and collector to the Stockport Infirmary in 1915, with full particulars of his career to date, we duly recorded in The Hospital of August 28, 1915, page 149, has been promoted to the post of General Superintendent and Secretary to the Royal Albert Edward Infirmary, Wigan. Mr. Lucas has held his post at Stockport for two years and a quarter. The past history and administration of the Royal Albert Edward Infirmary is full of interest, and reflects the greatest credit upon those who have been responsible for its efficiency. The post of Superintendent demands from its holder high character, integrity, and administrative gifts. In collecting money at Stockport Mr. Hedley Lucas has displayed considerable energy and success.

### SURGICAL APPLIANCES FOR SCHOOL CHILDREN

The Medical Department of the Board of Education is obviously anxious to secure that the medical treatment of school children should be complete, and it is now sanctioning expenditure for surgical appliances. Thus approval has been given to the West Ham Education Committee, which submitted a proposal for supplying surgical appliances in necessitous cases and for arranging, if possible, for medical and surgical treatment at hospitals. The Board has intimated that it does not desire to raise any objection to the provision of appliances for children attending ordinary public elementary schools in cases where the parents are unable to pay. The Board, however, asks for information regarding any cases in which it is proposed to arrange for medical or surgical treatment at hospitals.

### A SUCCESS IN NORTH STAFFORDSHIRE.

It is good to hear that the organisation of sectional subscriptions, particularly among the employers' associations of the neighbourhood, has already produced over £1,600 of new annual subscriptions. The associations of employees have been also eager to help, and, in view of the activity displayed by Major Basil Rhodes, it is hardly surprising to learn that the special appeal for £10,000, which has been carried on independently, has produced already £5,000 or more. This is the reward of energy, and should encourage all hospitals to pursue the methods which have borne such satisfactory fruit.

### THE BRADFORD INSTITUTE FOR THE BLIND.

There are few cities which study the welfare of the blind to the same extent that Bradford does. It was one of the first in the United Kingdom to grant free travel on the municipal tramways to the sightless—an example in which Halifax later followed suit—and now the Corporation has contributed £1,000 to the Bradford Institute for the Blind to enable a weekly war bonus to be paid to the inmates of the institution. It is pointed out that although the food and clothes of the blind cost very much more than in pre-war days, the earnings of sightless workers, always very small, have remained almost stationary.

### THE SALE OF BABIES.

At the recent annual meeting of the Association of Poor-Law Unions a resolution was considered urging that it should be an offence for mothers to sell their children for a lump sum. One speaker declared that many babies disappeared when they were a month old, and created the customary scare by asserting that babies were being sold to the wives of interned Germans. This, surely, is a reflection on our own race, and the conference apparently thought so, for the matter was referred back to the committee. Sir J. Spear, who presided, expressed a fear that an attempt would be made to abolish the Guardians, and urged that county and municipal bodies were already too busy to do Poor-Law work properly. The tendency to centralisation, however, continues to grow.

### A CHRISTMAS "GAZETTE."

The Christmas number of the *Gazette* of the 3rd London General Hospital is chiefly noteworthy for reminding the public of the exhibition now open at the Camera Club, 17 John Street, Adelphi. We give an account elsewhere of original black-and-white drawings from the *Gazette* which are exhibited at the club. They are well worth a visit. The Christmas number contains the text of a pantomime with a series of illustrations, and there are some excellent photographs of (presumably) last winter's snow. There is also an article by Mr. Pett Ridge, who is now librarian to the hospital. We must also mention the new cover design which Lance-Corporal J. H. Dowd has drawn for this number.

### THIS WEEK'S DRUG MARKET.

Business is by no means so quiet as it usually is at this period of the year; transactions, it is true, are confined to a small volume, but, considering the difficulties in the way of business and the extremely high cost of practically all drugs, it is really surprising that the market is as active as it is. Several price changes are to be noted. Makers of strychnine have again advanced their prices, mainly in consequence of the high cost of nux vomica. Quotations for codeine have been raised, and it seems very probable that makers of morphine will advance their prices; the nominal price of morphine has been the same for about a year, notwithstanding the large advance in the value of opium, and it is quite probable that when quotations for morphine are raised the rise will be substantial. Glucose is again dearer, but the slightly downward tendency in the price of saccharin is still noticeable. The upward tendency in the values of antifebrin, benzoic acid, benzoate of soda, hexamine and formaldehyde continues. Among the few drugs the prices of which are not firmly maintained are phenacetin and resorcin.

## Figures and Tables

**Figure f1:**